# Revolutionizing type 2 diabetes management: the role of the pharmacist in unlocking the potential of tirzepatide

**DOI:** 10.1007/s44446-025-00050-2

**Published:** 2025-12-17

**Authors:** David Banji, Saeed Alshahrani, Moaddey Alfarhan, Otilia J. F. Banji

**Affiliations:** 1 Department of Pharmacology & Toxicology, College of Pharmacy, Jazan University, Jazan, Saudi Arabia; 2 Departement of Clinical Practice, College of Pharmacy, Jazan University, Jazan, Saudi Arabia; 3 Pharmacy Practice Research Unit, College of Pharmacy, Jazan University, Jazan, Saudi Arabia

**Keywords:** Tirzepatide, Dual GIP/GLP-1 agonist, Type 2 diabetes mellitus, Weight loss therapy, Cardiometabolic health

## Abstract

Type 2 diabetes mellitus (T2D) affects over 460 million people worldwide and profoundly diminishes their quality of life. Its onset is driven by obesity, sedentary behavior, and genetic predisposition, leading to insulin resistance, progressive beta-cell dysfunction, and complications such as cardiovascular disease and nonalcoholic fatty liver disease (NAFLD). Effective management requires therapies that extend beyond glycemic control to address these interconnected risks. Tirzepatide, a dual agonist of glucose-dependent insulinotropic polypeptide (GIP) and glucagon-like peptide-1 (GLP-1) receptors, has demonstrated remarkable efficacy in clinical studies, particularly the SURPASS trials. It achieved a reduction in HbA1c of up to 2.3% and body weight losses exceeding 22% over 72 weeks. Additional benefits include improvement in blood pressure, lipid levels, and inflammatory markers, with potential benefit in NAFLD. Pharmacists play a vital role in translating these benefits into practice by counselling patients on safe use, managing adverse effects, and supporting adherence. Their involvement bridges the gap between trial evidence and real-world outcomes. This review examines the dual mechanism of tirzepatide, its broader metabolic effects, and the critical role of pharmacists in optimizing its use. With pharmacist-led care, tirzepatide has the potential to transform diabetes management across diverse populations.

## Introduction

Type 2 diabetes mellitus (T2D) accounts for over 90% of diabetes cases worldwide and is one of the most significant global health challenges of our time (Magliano and Boyko 2021). Over the years, the prevalence of T2D has risen sharply, driven by sedentary lifestyles, unhealthy dietary habits, and ageing populations (Khan et al. 2020a; Galicia-Garcia et al. 2020). In 2019 alone, diabetes accounted for 11.3% of global deaths, but in the Middle East, this figure is higher, with diabetes accounting for 16.2% of deaths in the region (Saeedi et al. 2020). Evidence suggests that T2D can be prevented or delayed, and remission may sometimes be possible (Magliano and Boyko 2021). Despite advances in pharmacotherapy, the management of T2D remains challenging due to its multifactorial pathophysiology, requiring interventions to extend beyond glycemic control to target comorbidities such as obesity, cardiovascular disease, and hypertension. Unfortunately, many available treatments come with trade-offs, including weight gain, insufficient cardiovascular protection, or limited efficacy in specific patient populations (Guo et al. 2023).

Incretin-based treatments, particularly glucagon-like peptide-1 (GLP-1) receptor agonists, have revolutionized diabetes management in recent years. These therapies work dually by lowering blood sugar levels and promoting weight loss, effectively addressing two significant challenges in diabetes management. Additionally, they support cardiovascular health, significantly improving patient outcomes (Zheng et al. 2024). GLP-1 receptor agonists enhance insulin secretion, suppress glucagon release, and slow gastric emptying, all of which contribute to better glucose control and reduced body weight. Despite these benefits, gastrointestinal side effects and variable patient response continue to limit their effectiveness (Sodhi et al. 2023).

Building on this foundation, tirzepatide, a novel therapeutic advancement, acts as a dual agonist of the glucose-dependent insulinotropic polypeptide (GIP) and GLP-1 receptors (Chavda et al. 2022). By this dual approach, it amplifies the benefits seen with GLP-1 agonists by targeting both incretin pathways (Rizvi and Rizzo 2022). The GIP component enhances beta cell function and lipid metabolism, while the GLP-1 component improves glycemic control and reduces appetite, resulting in significant weight loss.

Clinical trial evidence, particularly from the SURPASS studies, demonstrates that tirzepatide provides effective glycemic control while simultaneously addressing multiple metabolic risks, offering an advantage over existing therapies (Jastreboff et al. 2022a; Tsukamoto et al. 2024; Forzano et al. 2022). Findings suggest that tirzepatide promotes body weight reduction, improves lipid profiles, and is beneficial for patients with nonalcoholic fatty liver disease (NAFLD). Emerging evidence also suggests the potential to lower cardiovascular risks, highlighting its role in diabetes management (Epelde 2024; Nauck and D’Alessio 2022). However, translating these results into routine practice presents practical challenges, including managing adverse effects and ensuring sustained patient adherence.

Within this context, pharmacists play a pivotal role in bridging the gap between clinical evidence and real-world outcomes. As accessible healthcare providers, pharmacists educate patients, monitor therapeutic responses and adverse effects, and ensure safe use of medications. This is especially critical in Saudi Arabia, where pharmacists often bridge gaps in specialized care.

Accordingly, this review examines the dual mechanism of action and clinical efficacy of tirzepatide, with a particular focus on its benefits beyond glycemic control. The review also highlights the pivotal role of pharmacists in improving patient outcomes and diabetes care within the Saudi healthcare context. The insights presented aim to support the integration of tirzepatide into multidisciplinary treatment strategies, ultimately enhancing health outcomes in the region.

## Methods

### Literature search strategy

A comprehensive search of electronic databases, including PubMed, Scopus, Web of Science, and Google Scholar was performed. The literature search covered publications from January 2010 to December 2024, ensuring the inclusion of recent and relevant advances in incretin-based therapies and tirzepatide research. Keywords such as "Tirzepatide," "GIP/GLP-1 receptor agonist," "Type 2 diabetes mellitus," "glycemic control," "weight loss," and "cardiovascular outcomes" were used. Boolean operators were used to refine and broaden the search as needed.

### Eligibility criteria

In selecting studies for this review, we prioritized research that provided meaningful information on the efficacy, safety, and impact of tirzepatide on glycemic and non-glycemic outcomes, such as weight loss and cardiovascular health. Eligible studies included randomized controlled trials, systematic reviews, meta-analyses, cohort studies, and observational research, all published in English and focused on adult patients (≥ 18 years) diagnosed with T2D. The review excluded articles that were not available in the English language, lacked full-text access, or were limited to case reports, expert opinion, or conference abstracts. Studies involving nonhuman subjects or unrelated therapies, as well as those published before 2010 or with incomplete data, were also excluded.

### Study selection

The initial search identified 1,356 articles. After removing duplicates and carefully selecting titles and abstracts for relevance, 112 full-text articles were reviewed in detail. Following their thorough evaluation, 56 studies were deemed suitable for inclusion. These studies formed the foundation of this review, providing extensive data on the unique dual mechanism of action of tirzepatide, its effectiveness in addressing both glycemic and metabolic challenges, and its potential to transform diabetes care. Furthermore, this review emphasizes the practical importance of tirzepatide, particularly its integration into pharmacy-led care models, which play a crucial role in the management of T2D. By employing a rigorous and transparent selection process, this review delivers findings that are comprehensive and relevant to clinical practice, particularly in the context where diabetes care is a growing priority.

### Data synthesis

The included studies were synthesized through a narrative framework. Data were extracted with a focus on the intervention characteristics and clinical outcomes relevant to tirzepatide, with a particular emphasis on glycemic control, weight reduction, and cardiovascular outcomes. Safety data, including gastrointestinal and hypoglycemic risk, were also reviewed. Studies were grouped thematically based on efficacy outcomes (glycemic control, weight loss, metabolic markers), safety outcomes, and pharmacy-led interventions.

### Ethical considerations

Ethical approval was not required for this review, as it did not involve the collection of primary data. By synthesizing existing evidence, this review highlights the promising role of tirzepatide in redefining the therapeutic landscape of T2D, providing valuable information for future research and clinical applications.

## Tirzepatide and glycemic control: Mechanism of action

### Enhanced insulin secretion and suppression of glucagon

**Fig. 1:**
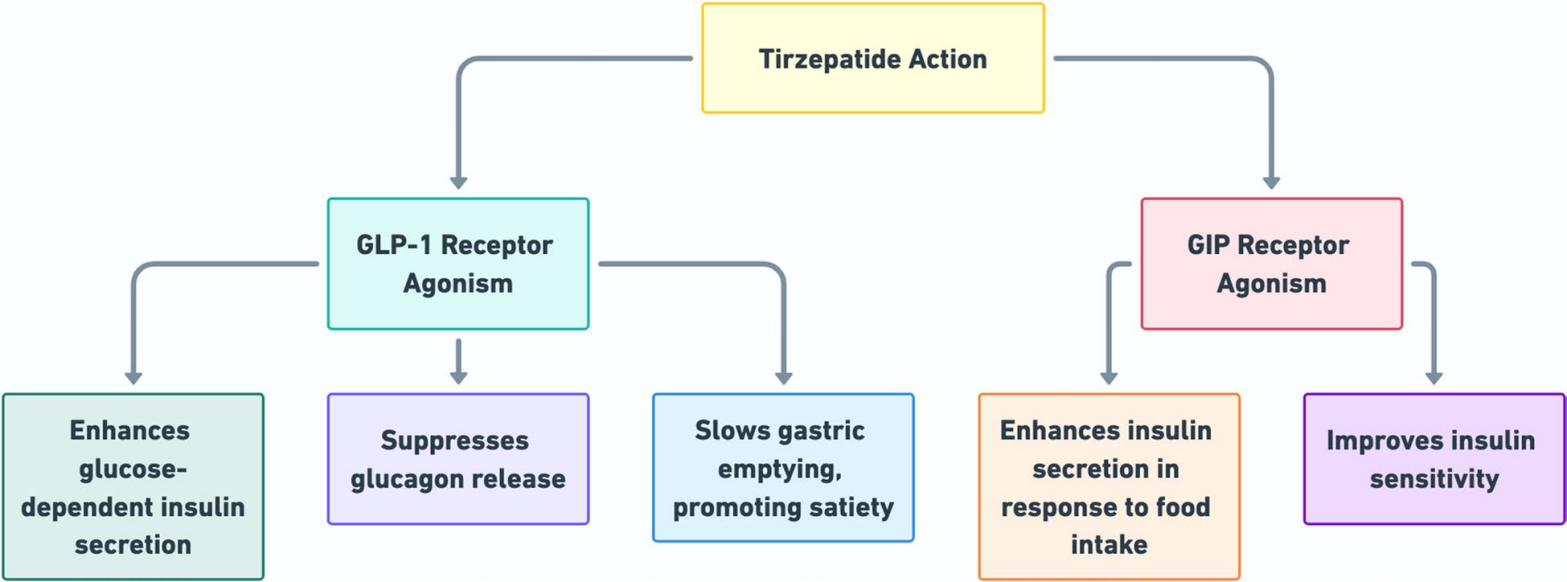
Flowchart summarizing the dual mechanism of action of tirzepatide

Tirzepatide harnesses the dual twincretin activity by acting through the GIP and GLP-1 receptors (MacIsaac et al. 2023), stimulating insulin secretion, suppressing glucagon release, regulating appetite, and increasing energy expenditure (Fig. 1). The role of GIP has historically been underestimated due to impaired receptor signaling, but it has been rediscovered as a powerful partner when paired with GLP-1. This multifaceted approach addresses blood sugar levels and tackles underlying metabolic challenges, making it a promising therapy for patients struggling with the complexities of T2D (Jastreboff et al. 2022a; Tsukamoto et al. 2024; Heise et al. 2023). Under hyperglycemic conditions, it amplifies the insulin response without increasing the risk of hypoglycemia, a common and serious concern with many traditional antidiabetic therapies (Liu 2024; Thomas et al. 2021).

Beyond its insulinotropic effects, the combined GIP and GLP-1 receptor activation influences glucagon regulation, contributing to further glycemic control. In addition, GIP plays a glucagonostatic role in hyperglycemic states, complementing the well-established ability of GLP-1 to suppress glucagon secretion. The dual mechanism reduces glucagon levels and decreases hepatic glucose production, thereby aiding in the overall management of T2D (Nauck and D’Alessio 2022; Al Zweihary 2024).

### Impact on appetite regulation and energy expenditure

Tirzepatide addresses another challenge associated with T2D, namely, weight management. GLP-1 receptor activation promotes satiety and reduces appetite by acting on the central nervous system, particularly the hypothalamus, the region regulating appetite, while slowing gastric emptying and prolonging post-meal satiety (Kabahizi et al. 2022). Tirzepatide helps increase feelings of fullness and suppress hunger signals, making it easier to eat less, while GIP receptor activation amplifies these effects by modulating fat metabolism and energy storage (Lopez-Mendez et al. 2023; Sinha et al. 2023). By targeting both the GIP and GLP-1 receptors, tirzepatide delivers complementary benefits, resulting in weight loss, which is essential for improving insulin sensitivity and overall metabolic health.

In addition to regulating appetite, tirzepatide modulates adipose tissue function by enhancing energy expenditure. GIP contributes to improving insulin sensitivity in adipose tissue, reducing ectopic fat accumulation, and optimizing lipid profiles. This action on both energy intake and expenditure has been observed in clinical studies, where participants reported less hunger and better appetite control (Jastreboff et al. 2022a; Jung and Jung 2022). However, long-term data from ongoing research and clinical trials will be needed to validate the benefits of tirzepatide further.

### Tirzepatide and glycemic control: evidence from clinical studies

The SURPASS program systematically evaluated tirzepatide across a range of clinical scenarios, from monotherapy to add-on therapy with oral agents and basal insulin. Across SURPASS-1 to SURPASS-6 trials, tirzepatide consistently achieved greater reductions in HbA1c (up to − 2.6%) and body weight (up to − 12.9 kg) compared with placebo, semaglutide, or insulin comparators (Rosenstock et al. 2023; Rosenstock, et al. 2020; Frias et al. 2021; Ludvik et al. 2021; Del Prato et al. 2021; Dahl et al. 2022). In addition, higher proportions of patients reached HbA1c < 7% on tirzepatide than on control treatments (Table 1). These outcomes highlight the ability of tirzepatide to improve glycemic control and promote weight loss across diverse patient populations.

**Table 1:** Key clinical trials of Tirzepatide with their strengths and limitations

Trial	Population/Treatment/comparator/Duration	HbA1c change (tirzepatide vs control)	Weight change (tirzepatide vs control)	Notes	Strengths	Limitation	Ref
SURPASS-1	T2D, Tirzepatide monotherapy (5, 10, or 15 mg)/Placebo/ 40 weeks	− 1.87% to − 2.07% vs ~ 0	− 7.0 to − 9.5 kg	Dose-dependent; first-line context	Double-blind, placebo-controlled design enhances validity	Early T2D, injectable-naïve, diet/exercise only subjects indicate selective early-stage population, which can reduce applicability Placebo-only comparator, a small sample (n≈478); and a duration of treatment of 40 weeks can limit generalizability	Jung and Jung (2022)
SURPASS-2	T2D/Tirzepatide (5, 10, or 15 mg)/Semaglutide (1 mg, once weekly as an add-on to metformin)/40 weeks	− 2.01%, 2.24% & − 2.30%or 5‐mg, 10‐mg, and 15‐mg doses of tirzepatide vs − 1.86%	Greater by 1.9–5.5 kg	Superior to semaglutide	Large, multinational cohort includes many patients across diverse countries and sites enhancing generalizability, while the large sample size increases statistical power, allowing even modest differences between treatments to be detected with confidence The trial employed an active comparator (semaglutide 1 mg), allowing fair benchmarking of tirzepatide against the standard of care. This design ensured clinically meaningful, real-world relevance and strengthened the credibility of the trial Use of a robust estimand framework (treatment regimen and efficacy estimands) minimized bias from treatment discontinuation or missing data, ensuring clinically meaningful results	The open-label design raises the risk of bias, especially when subjective outcomes like quality of life or side-effect burden are primary endpoints The study was restricted to patients on metformin only. In the management of T2D, many patients are on combination medications; hence, restricting to metformin might affect the external validity The 40-week duration is suitable to assess efficacy and safety signals, but there could be uncertainty about rare and long-term cardiovascular outcomes The comparator, semaglutide, was used in a dose of 1 mg and not the 2.4 mg dose used in clinical practice for the management of obesity High-risk individuals with proliferative or non-proliferative diabetic retinopathy, diabetic maculopathy, or recent acute myocardial infarction, stroke, or hospitalization for congestive heart failure were excluded. While this can reduce confounding and enhanced internal validity, it could also limit generalizability, as the findings may not apply to the multimorbid, high-risk patients commonly seen in clinical practice	Rosenstock et al. (2020)
SURPASS-3	T2D on oral agents/Tirzepatide (5, 10, or 15 mg)/Insulin degludec/52 weeks	− 1.93% to − 2.37% vs − 1.34%	− 7.5 to − 12.9 kg vs + 2.3 kg	82–93% vs 61% achieved HbA1c < 7%	The population included T2D patients on oral agents. The trial design ensured a fair and balanced comparator with degludec Conducted in a real-world context as an add-on to metformin ± SGLT2 inhibitors, the open-label design meant that both patients and physicians were aware of the assigned treatment, which could facilitate more accurate reporting of side effects	The open-label design has drawbacks; however, its use in a large trial spanning 13 countries and 122 sites was likely driven by logistical feasibility Moreover, the study excluded patients with complex comorbidities, which may limit the applicability of its findings to broader clinical practice Also, stable-weight requirement reduces representativeness	Ludvik et al. (2021)
SURPASS-4	T2D + increased CV risk/Tirzepatide (5 mg, 10 mg, or 15 mg)/Insulin glargine/ At least 52 weeks and a maximum of 104 weeks	− 2.43% to − 2.58% vs − 1.44%	− 7.1 to − 11.7 kg vs + 2.0 kg	High-risk population	The inclusion of a high–cardiovascular-risk T2D cohort makes the findings directly applicable to vulnerable patients, while the multinational design enhances generalizability across diverse healthcare settings A maximum treatment duration of 104 weeks allows assessment of glycemic control, weight effects, and CV safety signals Independent adjudication of CV events strengthens internal validity by reducing bias in outcome assessment	Open label design can lead to reporting and performance bias Trial results reflect head-to-head performance against basal glargine The trial was not powered as a formal CVOT, it could lack statistical rigor	Del Prato et al. (2021)
SURPASS-5	T2D on titrated glargine/Tirzepatide (5 mg, 10 mg, or 15 mg)/Placebo/ 40 weeks followed by a 4-week follow-up	− 2.34% to − 2.40% vs − 0.86%	− 5.4 to − 8.8 kg vs + 1.6 kg	Add-on to basal Insulin	Included T2D population on titrated basal glargine reflects real-world intensification of therapy and increases clinical relevance Double-masked trial reduces both patient and investigator bias in reporting outcomes Adjudication of safety endpoints increases accuracy and credibility	Placebo-only comparator (no active GLP-1 RA) Despite a 40-week treatment, the follow-up period was only 4 weeks, which may not be sufficient to identify long-term safety, renal, and cardiovascular outcomes Populations at the highest risk were excluded (BMI ≥ 23, CKD/retinopathy)	Dahl et al. (2022)
SURPASS-6	T2D on basal insulin/Tirzepatide (5 mg, 10 mg, or 15 mg)/Insulin lispro/ 72 weeks	− 2.1% vs − 1.1%	− 9.0 kg vs + 3.2 kg	68% vs 36% reached HbA1c < 7%	SURPASS-6 stands out because it compared tirzepatide head-to-head with prandial insulin lispro, the standard intensification step if basal Insulin fails. In real practice, clinicians faced with inadequate glycemic control on basal Insulin must decide between adding mealtime insulin or switching to a GLP-1 RA. By testing tirzepatide directly against lispro, the trial addresses a real-world decision of including lispro or tirzepatide The trial included patients who were inadequately controlled on basal Insulin, a common clinical scenario. As the design is identical to real-life practice, the results are generalizable Treatment for 52 weeks provides a robust assessment of HbA1c lowering, weight reduction, and safety, leaving behind the concern that the benefits are transient	Open label study can introduce bias Pooling multiple tirzepatide doses together increases statistical power but could obscure dose-specific effects The trial compared tirzepatide with prandial insulin lispro. Another real-world intensification option is basal Insulin plus a GLP-1 fixed-ratio combination. As this was not included, it could leave a gap in comparative effectiveness	Rosenstock et al. (2023)
SURMOUNT-1	Obesity (± T2D)/Tirzepatide (5 mg, 10 mg, or 15 mg)/Placebo/ 72 weeks	N/A	− 15.0%, − 19.5%, − 20.9% vs − 3.1%	Strong dose–response; cardiometabolic gains	Population included had obesity without diabetes Duration of study 72 weeks which is long enough trial to study obesity	As the SURMOUNT-1 trial enrolled relatively healthy participants and involved more intensive follow-up than usual care, its findings may not fully translate to real-world practice Moreover, a ≤ 2-year duration remains short for a chronic, relapsing disease like obesity, limiting long-term generalizability	Jastreboff et al. (2022b)
SURMOUNT-2	Obesity + T2D/Tirzepatide (10 mg or 15 mg)/Placebo/ 72 weeks	N/A	− 12.8% to − 14.7% vs − 3.2%	Effective in obese patients with diabetes	Included population with both obesity and type 2 diabetes over an extended follow-up, thus providing robust evidence on dual clinical benefits- substantial weight reduction along with improved glycemic control	Participants also received optimized diabetes care; hence, some benefits may reflect background management rather than the impact of tirzepatide alone, limiting generalizability to people with obesity but without diabetes	Garvey et al. (2023)
SURMOUNT-3	Obesity after lifestyle intervention/Tirzepatide (10 or 15 mg)/Placebo/ 72 weeks	N/A	− 18.4% vs + 2.5%	Add-on after an intensive lifestyle	Obesity patients after lifestyle run in A multicenter, double-masked, placebo-controlled study, which increased patient diversity and minimized bias, thereby enhancing external and internal validity The trial isolated the incremental benefit of tirzepatide on top of lifestyle change, strengthening internal validity	The trial did not use an active comparator, which could make it less pragmatic An enrichment design that selectively retains adherent, lifestyle-responsive participants could inflate the effect sizes, making the findings generalizable only to adherent, lifestyle-responsive patients	Wadden et al. (2023)
SURMOUNT-4	Obesity/Tirzepatide (10 or 15 mg)/Placebo/ 36-week open-label tirzepatide lead-in, then 52-week double-anonymized randomization to continue tirzepatide vs placebo	N/A	− 5.5% vs + 14.0%	Prevented weight regain after initial loss	Obesity maintenance trial, designed to test sustainability of weight loss 36-week run-in + 52-week randomized period providing long-term data SURMOUNT 4 is a multicenter, double-blind design, which enhances both internal validity and external generalizability, reducing bias while ensuring findings are applicable across diverse settings	Excluded early dropouts/non-responders, so less generalizable Could overestimate long-term maintenance under real-world adherence	Aronne et al. (2024)
SURMOUNT-5	Obesity but without type 2 diabetes mellitus/tirzepatide (10 mg or 15 mg)/semaglutide (1.7 mg or 2.4 mg)/72 weeks	NA	Tirzepatide: − 20.2% (95% CI, − 21.4 to − 19.1) Semaglutide: − 13.7% (95% CI, − 14.9 to − 12.6)		The trial enrolled adults with obesity but without diabetes and directly compared tirzepatide with semaglutide 2.4 mg, the established benchmark for obesity pharmacotherapy Also, the 72-week trial can provide robust, clinically relevant evidence	Excluding patients with type 2 diabetes and limiting follow-up to ~ 18 months restricts generalizability The absence of long-term cardiovascular outcomes leaves some uncertainty	Aronne et al. (2025)

## Tirzepatide dual mechanism: a paradigm shift beyond glycemic control

**Fig. 2:**
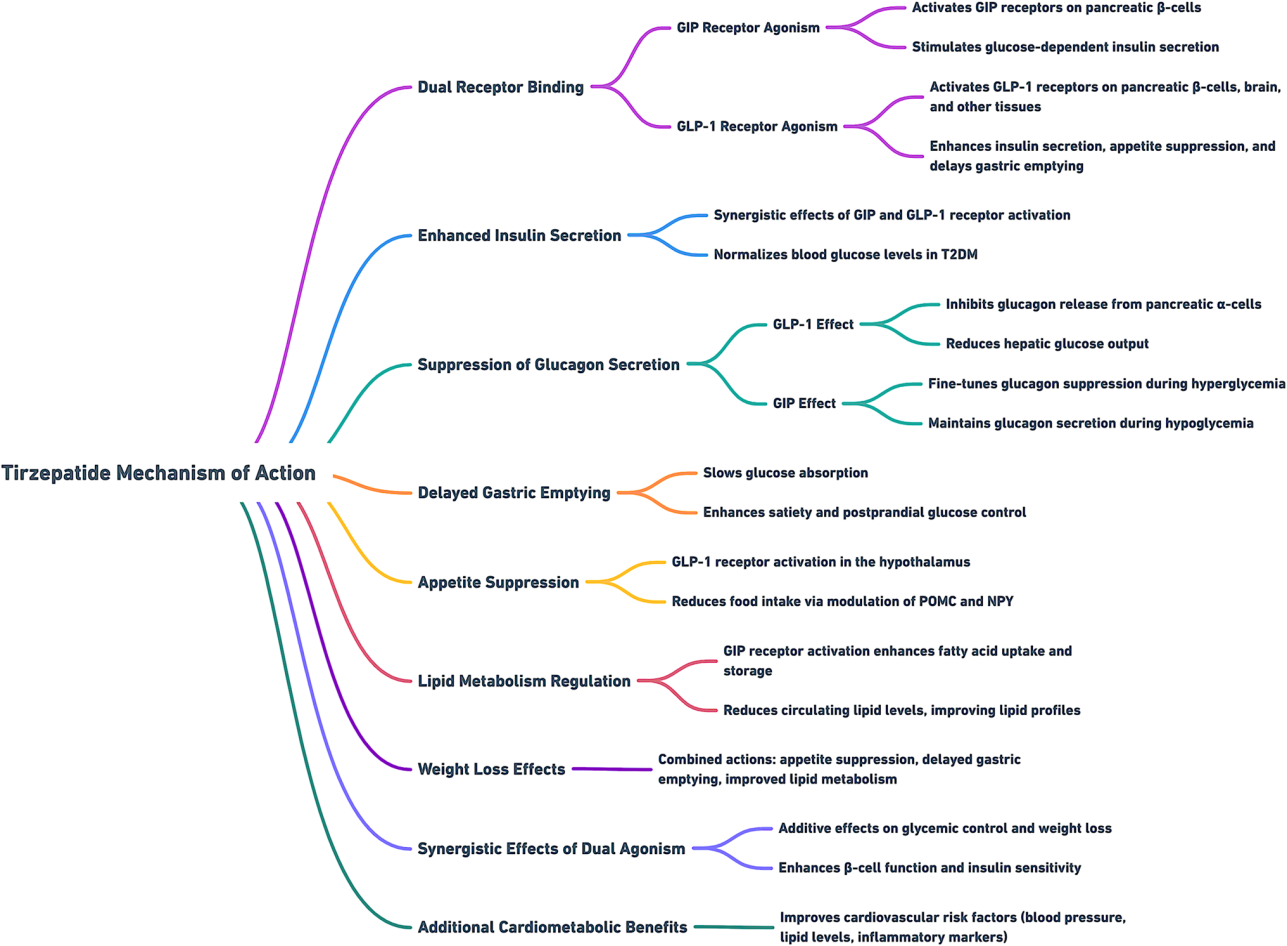
The mechanism of action of tirzepatide: A dual agonist of the glucose-dependent insulinotropic polypeptide (GIP) receptor and the glucagon-like peptide-1 (GLP-1) receptor. Its mechanism involves several physiological effects that improve glycemic control and promote weight loss

Tirzepatide aids in the management of metabolic challenges, including weight gain, lipid regulation, and cardiovascular health. These pleiotropic effects could position tirzepatide as an emerging therapeutic option for individuals with T2D and to address a broader spectrum of metabolic disorders (Fig. 2).

### Effects on weight management

**Fig. 3:**
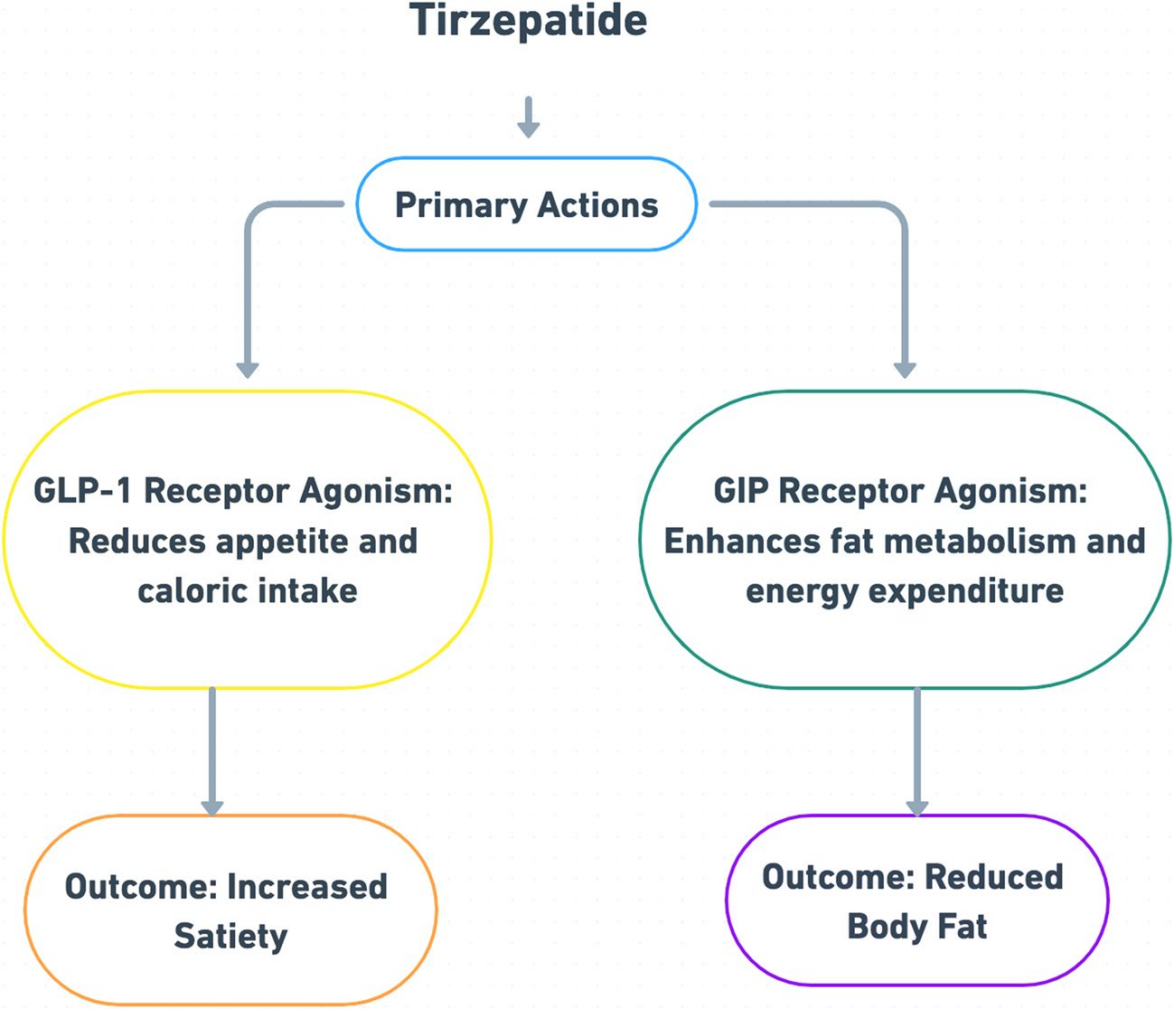
Mechanism of action of Tirzepatide on weight loss

The dual agonism of tirzepatide translates into clinically meaningful weight reduction by integrating GLP-1–mediated appetite suppression and delayed gastric emptying (Kabahizi et al. 2022) with GIP-driven improvements in lipid metabolism and energy expenditure (MacIsaac et al. 2023) (Fig. 3). This has been demonstrated in the SURMOUNT trials, where tirzepatide produced substantial body weight loss and improvements in cardiometabolic risk markers. Comparative analysis also indicated a greater proportion of patients achieving ≥ 10%−25% weight loss with tirzepatide than with semaglutide. Collectively, these findings show that tirzepatide produces weight loss at levels comparable to or greater than existing therapies, even in populations without T2D. The key outcomes from the SURMOUNT trials are summarized in Table 1.

### Lipid regulation

Tirzepatide has demonstrated efficacy in improving lipid profiles through GIP receptor activation, which increases the lipid buffering capacity of fatty tissue, facilitating triglyceride clearance and reducing ectopic fat accumulation. Concurrently, activation of the GLP-1 receptors improves lipid profiles, lowering low-density lipoprotein (LDL) cholesterol and triglyceride levels. Together, these effects work to reduce atherogenic risks, making tirzepatide a valid therapeutic option to manage dyslipidemia (Corrao et al. 2024).

Additionally, tirzepatide enhances energy expenditure and modulates adipose tissue function through its dual receptor-binding action. GIP signaling enhances insulin sensitivity, reduces lipolysis, and supports lipogenesis in metabolically active white adipose tissue. Meanwhile, activation of the GLP-1 receptors complements these effects by reducing visceral fat and optimizing the overall energy balance in the body. Together, these mechanisms contribute to a healthier metabolic profile, alleviating the burden of insulin resistance and reducing ectopic fat accumulation.

### Cardiovascular benefits

Observational and interventional studies have reported the influence of tirzepatide on cardiovascular health. Research indicates that tirzepatide enhances lipid clearance through its actions on GIP receptors (Liu 2024). Clinical trials further demonstrate its ability to reduce blood pressure, inflammatory biomarkers, and lipid profiles. The GIP components contribute to improving insulin sensitivity in cardiovascular tissues. At the same time, activation of the GLP-1 receptor reduces arterial stiffness and supports endothelial function, both of which are critical factors for cardiovascular well-being (Mori et al. 2020; Taktaz et al. 2024). It also reduces fat buildup around vital organs by activating GLP-1 receptors, contributing to a healthier heart.

An observational cohort study involving 47,719 adults (753 treated with tirzepatide and 46,966 on GLP-1RA) reinforced the cardio-renal benefits of tirzepatide. The primary composite outcome, which was acute myocardial infarction (AMI), ischemic stroke, and all-cause mortality, was significantly reduced in the tirzepatide group, with a relative risk reduction (RRR) of 40% (hazard ratio [HR]: 0.60; 95% confidence interval [CI]: 0.42–0.84; *P* = 0.003). Tirzepatide use was associated with a significant reduction in AMI (RRR: 42%, HR: 0.59; 95% CI: 0.38–0.91; *P* = 0.016) and all-cause mortality (RRR: 44%, HR: 0.35; 95% CI: 0.14–0.89; *P* = 0.021), while the reduction in ischemic stroke did not reach statistical significance. Clinically relevant secondary endpoints were also lowered, including heart failure events (RRR: 40%, HR: 0.60; *P* = 0.040), new-onset systolic heart failure (RRR: 26%, HR: 0.73; *P* = 0.045), atrial fibrillation/flutter (RRR: 45%, HR: 0.23; *P* = 0.004), and acute kidney injury (RRR: 33%, HR: 0.67; *P* = 0.028). These findings point to the benefits of tirzepatide in reducing cardiometabolic risk (Dani et al. 2025).

### Effects on nonalcoholic fatty liver disease

Tirzepatide targets multiple pathways related to NAFLD and nonalcoholic steatohepatitis (NASH), including hepatic fat accumulation, inflammation, and liver fibrosis (Heyens et al. 2021; Yang and Wang 2023). GIP receptor activation enhances hepatic lipid metabolism, while GLP-1 receptor agonism decreases hepatic glucose production and fat deposition. Evidence from clinical studies suggests that tirzepatide reduces key inflammatory markers such as CRP and IL-6, both of which are implicated in hepatic injury (Hartman et al. 2020). These changes attenuate fibrosis progression and scarring in NASH, lowering the risk of cirrhosis or other liver complications (Sanyal et al. 2024).

Improvement in body weight, lipid profiles, and glycemic levels observed with tirzepatide treatment may indirectly benefit liver health. Early clinical studies have indicated a reduction in liver stiffness, a surrogate marker of fibrosis, as well as improvement in multiple metabolic measures, but confirmatory evidence from extensive, long-duration studies is still required.

### Anti-inflammatory effects

Chronic inflammation is a crucial driver in the progression of metabolic syndrome and its associated complications. Clinical studies have shown that tirzepatide controls inflammation by significantly lowering inflammatory markers, such as high-sensitivity C-reactive protein (hsCRP) and IL-6, which are key contributors to cardiovascular disease. In the SURMOUNT-1 and −2 trials, tirzepatide significantly reduced inflammatory markers IL-6 and hsCRP over 72 weeks, but this was not observed with treatment for 24 weeks. Tirzepatide reduces inflammation primarily through weight loss, and this effect could be beneficial in overweight, obese individuals and those with metabolic dysfunction (Sattar et al. 2024).

## Renal protection

### Effects on albuminuria and kidney function

Tirzepatide improves renal health, particularly in individuals with T2D, who are at high risk of developing chronic kidney disease (CKD). Diabetic nephropathy, a common cause of CKD, is often marked by albuminuria and a gradual decline in kidney function. Research has shown that tirzepatide significantly reduces albuminuria, especially in patients with early or advanced signs of kidney damage (Sattar et al. 2024; Bosch et al. 2023). Tirzepatide was found to slow the decline in estimated glomerular filtration rate (eGFR), a key measure of kidney health, and did not increase the urine albumin-creatinine ratio (UACR) (Heerspink et al. 2022).

**Fig. 4:**
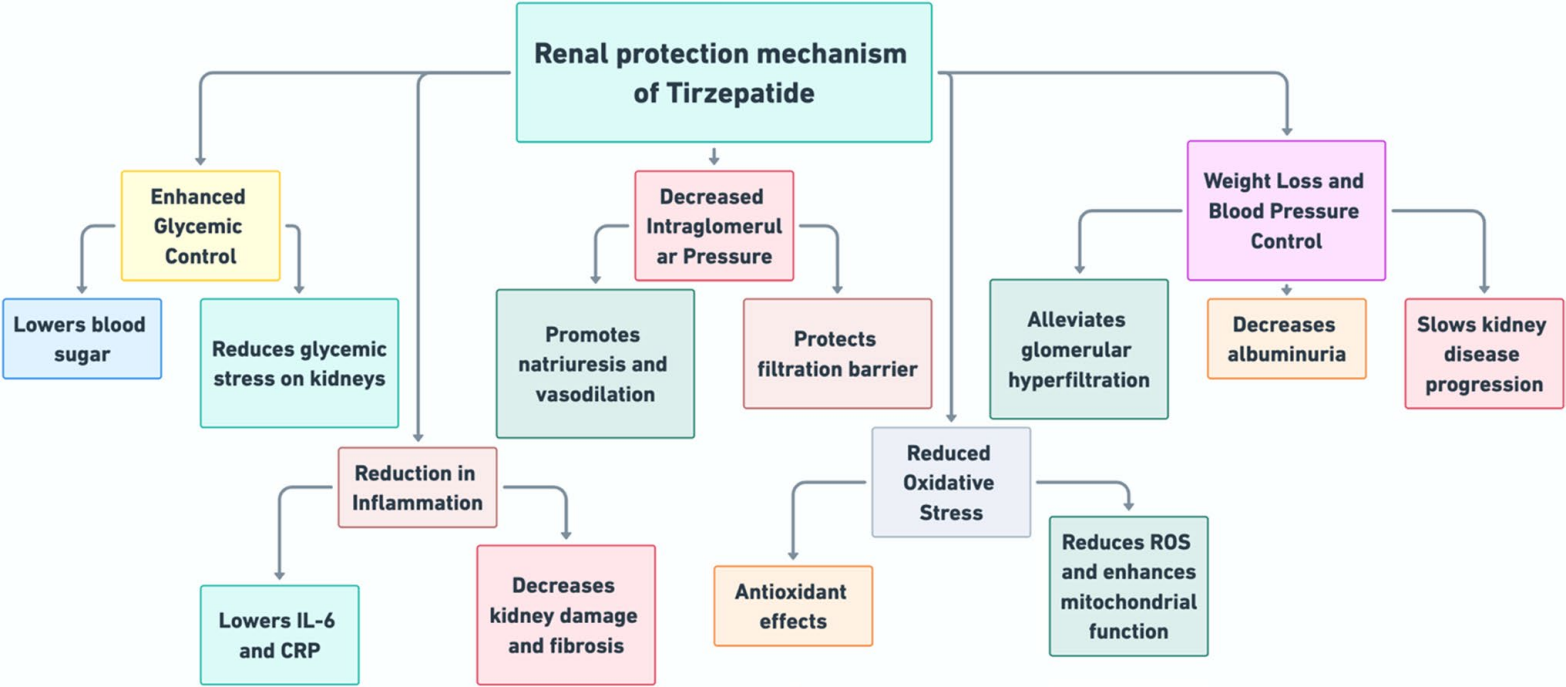
Mechanism of renal protection of Tirzepatide

The renoprotective effects of tirzepatide are mediated through multiple mechanisms. Firstly, it enhances insulin secretion and reduces glucagon levels, thereby controlling hyperglycemia and alleviating one of the primary contributors to renal damage (Nauck and D’Alessio 2022) (Fig. 4). It reduces systemic inflammation by lowering markers such as IL-6 and CRP, protecting the kidneys from inflammation-related damage and scarring (Bosch et al. 2023; Ho and Shirakawa 2022). Tirzepatide also improves renal hemodynamics by promoting natriuresis and vasodilation, which lowers intraglomerular pressure and preserves the integrity of the glomerular filtration barrier (Urva et al. 2021). Further, it combats oxidative stress, a critical factor in diabetic kidney disease, by enhancing mitochondrial function and reducing the formation of reactive oxygen species (ROS) (Yang et al. 2025). Its ability to promote weight loss and lower blood pressure can result in reduced hemodynamic stress on the kidneys, contributing to reduced albuminuria and slowing CKD progression (Kanbay et al. 2023).

### Metabolic syndrome: Possible role

Metabolic syndrome is a severe condition that combines central obesity, high blood pressure, dyslipidemia, and insulin resistance, which collectively increase the risk of heart disease and T2D. Evidence suggests that tirzepatide might target multiple components of this syndrome through dual agonism of GIP and GLP-1 receptors, including improvement of lipid profiles and reducing ectopic fat accumulation, particularly visceral fat (Kanbay et al. 2023). These changes have been associated with enhanced insulin sensitivity, potentially lowering cardiovascular risk. Additionally, tirzepatide also reduces both systolic and diastolic blood pressure and improves vascular function. As inflammation is a key driver of metabolic syndrome, reducing inflammatory markers could help alleviate the disease burden.

## Safety and tolerability

### Adverse events reported in clinical trials

Tirzepatide offers therapeutic benefits and demonstrates a safety profile comparable to that of other incretin-based therapies, such as semaglutide and dulaglutide (Jung and Jung 2022). Consistent with this class, the most commonly reported adverse effects include nausea, vomiting, and diarrhea, especially at higher doses (Table 2) (Dahl et al. 2022; Rosenstock et al. 2023; Jastreboff et al. 2022b).

**Table 2:** Adverse drug reactions of Tirzepatide

System	Adverse Reactions/Side Effects	Frequency	References
Gastrointestinal	Nausea, diarrhea, vomiting, constipation, abdominal pain, dyspepsia, gastroesophageal reflux disease, abdominal distension, Cholelithiasis, acute pancreatitis, increased serum pancreatic amylase and lipase levels, acute gallbladder disease	Very common (≥ 10%)	Rosenstock et al. (2023); Ludvik et al. (2021); Del Prato et al. (2021); Dahl et al. (2022)
Cardiovascular	Sinus tachycardia, increased heart rate	Very common (≥ 10%)	Tirzepatide Side Effects (n.d.)
Metabolic	Hypoglycemia (especially when used with insulin or sulfonylureas)	Common (1% to 10%)	Tirzepatide Side Effects (n.d.)
Dermatological	Injection site reactions (e.g., redness, itching)	Less common (< 10%)	Tirzepatide Side Effects (n.d.)
Endocrine	Potential risk of thyroid C-cell tumors (observed in animal studies), pancreatitis, hypoglycemia	Not determined	Abi Zeid Daou et al. (2025); Madsen et al. (2012)
Ophthalmological	Diabetic retinopathy complications	Not determined	Tirzepatide Side Effects (n.d.)
Respiratory	Risk of aspiration during anesthesia due to delayed gastric emptying	Not determined	Jalleh et al. (2024)

Lower Dose (5 mg/week): Better tolerability with fewer gastrointestinal adverse effects but reduced efficacy in glycemic control and weight loss. Moderate Dose (10 mg/week): Balanced efficacy and adverse effects

High Dose (15 mg/week): Maximum efficacy but with increased adverse events, particularly GIAEs and discontinuations

While its dual GIP/GLP-1 agonism may marginally increase the frequency of these issues compared to other GLP-1 monotherapies, these events are mild and manageable. The risk of hypoglycemia with tirzepatide remains low, consistent with other GLP-1 receptor agonists, particularly in the absence of concomitant Insulin or sulfonylureas (Frias et al. 2021). The side effects that appear during early treatment, especially at higher doses, are transient and resolve as patients adjust to the medication (Tables 3 and 4).

**Table 3:** Dose-dependent adverse effects of Tirzepatide

Adverse Effect	Description	Dose-Dependent Trend	Typical Doses	Source
Hypoglycemia	Lower risk compared to basal insulin but can occur when combined with other agents	Minimal but present; risk slightly increases at higher doses	Occurs primarily at 10–15 mg weekly	Rosenstock et al. (2023); Frias et al. (2021); Ludvik et al. (2021); Del Prato et al. (2021); Dahl et al. (2022)
Discontinuation Rates	Patients discontinuing due to adverse effects, particularly GIAEs	Higher doses correlate with increased discontinuation rates	Discontinuation higher with 10–15 mg weekly	Garvey et al. (2023); Jastreboff et al. (2022b)
Blood Pressure	Reduction in systolic and diastolic blood pressure	Greater reductions observed with higher doses, necessitating cardiovascular monitoring	Most pronounced at 10–15 mg weekly	Kanbay et al. (2023)
Weight Loss	Significant weight reduction is beneficial but requires caution for metabolic stability	Greater weight loss is achieved at higher doses	Significant weight loss at 10–15 mg weekly	Aronne et al. (2025); Jastreboff et al. (2022b); Garvey et al. (2023); Wadden et al. (2023); Aronne et al. (2024)

**Table 4:** Benefits and associated risks of Tirzepatide

Benefits	Risks
**Improved Glycemic Control**: Significantly lowers blood glucose levels in individuals with type 2 diabetes (Ludvik et al. 2021; Rosenstock, et al. 2020; Frias et al. 2021)	**Gastrointestinal Issues**: Common side effects include nausea, vomiting, diarrhoea, decreased appetite, constipation, and abdominal discomfort (Ludvik et al. 2021; Rosenstock, et al. 2020; Frias et al. 2021)
**Weight Reduction**: Leads to substantial weight loss, beneficial for overweight or obese patients (Wadden et al. 2023; Jastreboff et al. 2022b; Garvey et al. 2023)	**Pancreatitis**: There is a risk of developing inflammation of the pancreas (Tirzepatide Side Effects n.d.)
**Cardiovascular Benefits**: May reduce the risk of major adverse cardiovascular events and improve heart failure outcomes (Dani et al. 2025; Taktaz et al. 2024; Mori et al. 2020)	**Thyroid Tumors**: Potential risk of thyroid C-cell tumors, including medullary thyroid carcinoma (Abi Zeid Daou et al. 2025; Tirzepatide Side Effects, n.d.; Madsen et al. 2012)
**Blood Pressure Reduction**: Associated with decreases in systolic and diastolic blood pressure (Kanbay et al. 2023)	**Hypoglycemia**: Increased risk when used with other antidiabetic medications, particularly sulfonylureas or insulin (Lingvay et al. 2023)
**Renal Protection**: May offer benefits in reducing the progression of diabetic kidney disease (Heerspink et al. 2022; Bosch et al. 2023)	**Gallbladder Disease**: Potential risk of gallbladder-related issues (Zeng et al. 2023)

Some also experience minor injection site reactions or slight changes in certain enzyme levels, such as amylase and lipase (Ludvik et al. 2021).

### Cost-effectiveness of Tirzepatide

The dual action of tirzepatide reduces the reliance on multiple medications, alleviating both the financial and clinical burden faced by patients and the healthcare system (Zhang and McAdam Marx 2023). By effectively decreasing HbA1c levels and supporting weight loss, tirzepatide aids in preventing cardiovascular complications, nephrotoxicity, and neurotoxicity, which are key contributors to recurrent hospitalization and escalating healthcare costs. Although the upfront cost may be higher, its long-term clinical benefits and cost savings could make it a valuable investment.

### Patient-reported outcomes and quality of life

Managing diabetes can be overwhelming, and many patients face physical, emotional, and practical challenges. For individuals dealing with obesity alongside diabetes, the ability of tirzepatide to promote significant weight loss is transformative. Weight loss can boost self-confidence, reduce stigma, and make daily activities manageable. Tirzepatide has been shown to improve the quality of life for patients (Gregg et al. 2023; Boye et al. 2024), enhance functional ability, and increase physical mobility, enabling patients to gain control over their health (Lisco et al. 2024).

### Safety information for proper use

Despite improvements in the quality of life, ensuring the safe and effective use of the medication can be achieved through a few simple, yet crucial steps. The process begins carefully by reading the medication guide or instructions, which outline the safe use, side effects, and precautions to avoid complications. Patients must follow the prescribed dosage and schedule and consult the physician if any dose adjustments are needed. Recommended subcutaneous sites include the abdomen, upper thighs, or the back of the upper arms. Rotating the injection sites with each dose, rather than using the same location repeatedly, can help minimize tissue irritation.

For once-weekly medications like tirzepatide, selecting a consistent day and time each week can enhance adherence. Simple reminders, such as phone alarms or calendar alerts, can help patients stay on track with their medication schedule (Ahmad and Joshi 2023).

In the case of an accidental overdose, it is essential to remain composed and seek expert guidance by contacting a healthcare provider or a poison control center.

Starting a new injectable medication can be overwhelming, but taking the proper steps can make the process smoother and safer. First, receiving hands-on training from a healthcare provider, including demonstration and supervised practice, ensures that the medication is being used correctly and builds confidence.

Patients should also disclose all medications and supplements currently being taken, including prescription, over-the-counter drugs, vitamins, and herbal remedies, to avoid unexpected interactions and optimize therapeutic efficacy. For individuals who manage diabetes with Insulin or sulfonylurea, dose adjustments might be needed to reduce the risk of hypoglycemia (Cross et al. 2020). Underlying medical conditions, such as pancreatic dysfunction, renal impairment, or gastrointestinal disorders like gastroparesis, can significantly impact the pharmacokinetics and pharmacodynamics of prescribed medications. Open communication regarding these health issues enables healthcare professionals to strategize their therapeutic approaches, thereby mitigating potential risks and enhancing the overall efficacy of treatment interventions.

Individuals on oral contraceptives must be informed that tirzepatide may transiently reduce the effectiveness of contraceptive therapy (Skelley et al. 2024). It is advisable to discuss this with their healthcare provider, who may recommend alternative methods during the first few weeks of treatment or after any dosage changes. Similarly, individuals planning a pregnancy, currently pregnant, or breastfeeding should consult with their healthcare provider as tirzepatide may not be a suitable option, and adjustments may be needed to ensure the safety of the parent and the child (Skelley et al. 2024).

Patients with a history of diabetic eye issues, such as diabetic retinopathy, should communicate this information to their provider. Evidence suggests that tirzepatide may exacerbate diabetic retinopathy in some individuals, so early disclosure allows for appropriate monitoring or the consideration of alternative treatment options, if necessary (MacIsaac et al. 2023).

By following these steps, the treatment process can be efficient, safe, and effective. Moreover, this would foster a collaborative relationship with the healthcare provider and support a patient-centered approach to care (Table 5).

**Table 5:** The safety and usage instructions for Tirzepatide

Category	Details
**Safety Information**	
Familiarize yourself	Read the "Instructions for Use" booklet for essential safety details
Follow provider's advice	Use tirzepatide as directed by your physician and avoid dosage adjustments without consulting them
Injection sites	Subcutaneous injection in the stomach (abdomen), thigh, or back of the upper arm
Weekly routine	Administer tirzepatide once a week at any convenient time, with reminders or calendar marks to ensure consistency
Rotate injection sites	Change the injection site weekly to prevent irritation or complications
Overdose	In case of overdose, immediately contact your healthcare provider or Poison Control Center for assistance
**Important Tips**	
Learn proper usage	Healthcare providers will guide and demonstrate the correct administration technique
Discuss current medications	Inform your provider if you use insulin or sulfonylureas to avoid hypoglycemia. Recognize symptoms like dizziness, sweating, and shakiness
Birth control adjustments	Oral contraceptives may be less effective. Consider alternative methods for the first 4 weeks after starting tirzepatide or any dose increases
**Questions for Provider**	
Medical history	Share any history of pancreas/kidney problems, gastroparesis, or digestion issues
Current diabetes treatment	Mention if using medications like insulin or sulfonylureas, as dosage adjustments may be needed to prevent hypoglycemia
Vision concerns	Inform about diabetic retinopathy; tirzepatide may impact eye health, requiring monitoring
Medications and supplements	Disclose all prescription drugs, over-the-counter medicines, vitamins, and herbal supplements to avoid harmful interactions
Pregnancy and breastfeeding	Inform if pregnant, planning pregnancy, or breastfeeding. Tirzepatide may harm the unborn baby, and its safety in breastfeeding is uncertain. Discuss with the provider to decide the best approach
**Warnings**	
Thyroid tumor risk	Tirzepatide may increase thyroid tumor risk. Watch for signs such as a neck lump, swelling, hoarseness, difficulty swallowing, or shortness of breath. Consult a provider if these symptoms occur
Avoid use of if-	History of medullary thyroid carcinoma (MTC), Multiple Endocrine Neoplasia syndrome type 2 (MEN 2), or previous allergic reaction to tirzepatide. Allergic reaction symptoms include rash, itching, severe dizziness, or difficulty breathing

### Potential risks of Tirzepatide

Tirzepatide may carry specific risks, including a potential link to thyroid tumours or thyroid cancer (Lingvay et al. 2023; Zeng et al. 2023). Although these risks are rare, staying vigilant is crucial. Symptoms such as unusual neck lumps, ongoing hoarseness, difficulty in swallowing, or shortness of breath might indicate thyroid problems. Prompt medical attention is essential if these signs appear, ensuring early evaluation.

Some medications are not recommended for individuals with certain medical conditions. For example, individuals with a history of medium-sized thyroid carcinoma (MTC) or those diagnosed with Multiple Endocrine Neoplasia Syndrome type 2 (MEN 2) should avoid specific therapies (Abi Zeid Daou et al. 2025; Zeng et al. 2023). Sharing a detailed medical history with a healthcare provider enables them to select treatments that are most appropriate for individual needs. A disproportionality analysis of the FDA Adverse Event Reporting System (FAERS) data from 2004 to Q1 2024 revealed significant associations between GLP-1 receptor agonists and reported cases of thyroid cancer. The analysis revealed elevated reporting odds ratios (ROR) for tirzepatide (ROR = 2.09; 95% CI: 1.51–2.89), indicating a potential signal that warrants further investigation (Khurana et al. 2024).

Reports of acute pancreatitis, though rare, have emerged in trials and post-marketing settings (Zeng et al. 2023). Furthermore, severe allergic reactions to tirzepatide should not be overlooked (Tirzepatide Side Effects n.d.). Past reactions, such as rashes, severe dizziness, or breathing difficulties, should be disclosed to the healthcare providers. If such symptoms occur while on treatment, immediate medical assistance is crucial to address potential complications. These precautions are not intended to cause concern, but to support informed and proactive health management (Table 5).

## Pharmacist's role in the use of Tirzepatide

Pharmacists contribute to the effective use of tirzepatide by applying interventions that link its mechanisms of action to measurable clinical outcomes. At the initiation of therapy, pharmacists provide instruction on injection techniques and counselling to address patient concerns about the use of a once-weekly injectable. These measures help patients develop confidence, improve administration accuracy, and may reduce early discontinuation. Pharmacists also support dose titration by arranging follow-up visits, either in person or through telehealth. Such follow-up allows timely adjustment of therapy and helps manage gastrointestinal effects that are frequently associated with GLP-1 receptor activation. Practical advice, such as consuming small, low-fat meals, has been shown to reduce nausea and vomiting, thereby supporting continued treatment.

Pharmacists also play an essential role in safety monitoring. Regular evaluation of HbA1c, body weight, and cardiovascular parameters enables early detection of inadequate response or emerging risks. Since tirzepatide enhances insulin secretion and suppresses glucagon, concurrent use with sulfonylureas or Insulin may increase the risk of hypoglycemia. Pharmacist-led medication reviews and dose adjustments can reduce this risk and contribute to safer glycemic control. In addition, by monitoring renal indices such as estimated glomerular filtration rate (eGFR) and urine albumin-to-creatinine ratio, pharmacists can detect early changes in kidney function and collaborate with physicians to adapt therapy.

Evidence supports the clinical value of these contributions. A meta-analysis of 35 studies involving 4,827 patients found that pharmacist involvement resulted in significant improvements in clinical outcomes. Pharmacist-led care resulted in a mean reduction in HbA1c of 0.70%, along with improvements in medication adherence (Zhang et al. 2024). In a separate systematic review and meta-analysis, pharmacist interventions were compared with usual care and found to significantly reduce HbA1c levels by 0.85% (95% CI: −1.06 to −0.65; *P* < 0.0001), despite moderate heterogeneity (I^2^ = 67.3%) (Aguiar et al. 2016).

Adherence support is another area where pharmacists provide value. Techniques such as motivational interviewing and the use of digital reminders are beneficial for once-weekly dosing regimens. These strategies help maintain consistent use of tirzepatide and extend the durability of its metabolic benefits, including appetite suppression and weight reduction. Sustained weight loss, when combined with dietary and lifestyle counselling, has downstream effects on blood pressure, lipid profiles, and systemic inflammation.

With the increasing use of GLP-1 RAs in the management of T2D, regular follow-up is crucial to optimize dosing and prevent clinical inertia. To address these needs, a pharmacist-led GLP-1 RA titration service within an ambulatory care setting led to a significant reduction in mean HbA1c by 1.8% (*P* < 0.001) within 3 to 6 months post-titration. In addition, patients experienced an average weight loss of 8.1 kg (*N* = 107; *P* < 0.001), and the average number of diabetic medications was reduced from 2.5 to 2.1. These outcomes highlight the significant role of ambulatory care pharmacists in managing GLP-1 RA therapy (Miller et al. 2025).

In another single-center, prospective study conducted within a cardiology clinic, a pharmacist-led weight management service incorporating GLP-1 receptor agonist therapy with lifestyle counselling yielded an average weight loss of 12.6% over six months among 31 patients. Other benefits included a reduction in glycated hemoglobin (HbA1c) by 0.6%, low-density lipoprotein (LDL) cholesterol by 18 mg/dL, triglycerides by 29 mg/dL, systolic blood pressure by 9 mm Hg, and diastolic blood pressure by 2 mm Hg, demonstrating the benefits of pharmacists' involvement (Yates et al. 2024). These findings emphasize the vital role of pharmacists in contributing meaningfully to chronic disease management.

Pharmacists can play an essential role in optimizing patient outcomes through a multifaceted approach. They provide education on treatment benefits, side effects such as gastrointestinal discomfort or weight management expectations, thereby fostering trust and improving adherence. Practical guidance, such as dietary modifications, can help mitigate gastrointestinal discomfort, leading to uninterrupted treatment. Beyond education, pharmacists contribute to closely monitoring treatment efficacy and safety by assessing key metrics such as HbA1c levels, weight changes, and cardiovascular markers (Miller et al. 2025). The monitoring can help to identify individual variability in response to tirzepatide and collaborate with physicians to tailor treatment plans. Pharmacists play a crucial role in medication management by preventing drug-drug interactions, thereby enhancing patient safety. Furthermore, by promoting lifestyle changes, they could amplify the benefits of tirzepatide on glycemic control and cardiometabolic health (Bukhsh et al. 2018). Through regular follow-ups and motivational counselling, they can promote long-term adherence, addressing barriers such as treatment costs or misconceptions about therapy.

### Regional context: Saudi Arabia and the Gulf

In the Gulf region, particularly in Saudi Arabia, pharmacists can play a vital role in translating innovative therapies, such as tirzepatide, into improved real-world patient outcomes (Carris et al. 2023). With more than 4.27 million people affected by diabetes in Saudi Arabia (Diabetes in Saudi Arabia (2021), pharmacists' contributions are central to managing this public health challenge. Incorporating tirzepatide into diabetes care requires a multidisciplinary approach where pharmacists can play a key role in providing education, medication management, and personalized patient support.

The Saudi healthcare system, however, presents some structural barriers that complicate the integration. Care delivery remains centralized in major cities and is heavily focused on secondary and tertiary services, while community pharmacists remain underutilized in chronic disease management pathways. This restricts their potential to contribute to early diabetes management. In addition, the transition from a government-sponsored model to an insurance-driven, market-oriented system has introduced uncertainty in both funding and service coverage (Al-Arifi 2014). Newer therapies such as GLP-1 receptor agonists and dual GIP/GLP-1 agents are prohibitively expensive, raising concerns about long-term sustainability and limiting patient access. While extended services such as pharmacist-led weight-management programs or counseling can bridge the gap, they are offered at an additional payment, which can deter patients from utilizing these services (Alharthi 2024).

Logistical and workforce barriers also constrain pharmacist-led care. Shortages in personnel, insufficient private consultation space, limited time for individualized counselling, and inadequate equipment could restrict the scope of their services (Alharthi 2024). Even when pharmacists are trained and motivated, low health literacy and cultural sensitivities in some populations can reduce the impact of interventions. Despite this, strong evidence from the Middle East shows that pharmacist interventions can improve diabetes knowledge, self-care, and medication adherence among patients with T2D (Yates et al. 2024; Zhang et al. 2024; Aguiar et al. 2016; Miller et al. 2025; Bakhsh 2025). A few Saudi-based studies reinforce the value of pharmacists in diabetes care. One pharmacist-led program in Riyadh involved adult patients who attended at least three follow-up visits over nine months. At baseline, the mean HbA1c was 9.5% ± 1.3%, with 13 patients having higher levels (≥ 10%). By the end of the study, there was a significant drop in HbA1c levels to 8.3% ± 1.4% (*p* = 0.0004), with nine patients achieving a target HbA1c of < 7%. The study demonstrated that pharmacist-led interventions such as medication reviews, adherence counselling, and lifestyle education contributed to glycemic control (Alsuwayni and Alhossan 2020). Another initiative involving community pharmacists resulted in a 25% improvement in adherence rates, achieved through frequent follow-ups and educational sessions (Khan et al. 2020b). Similar efforts have been undertaken in other GCC countries, such as the national awareness campaigns of the Qatar Diabetes Association, which emphasize the role of pharmacists in public education about diabetes management (El Hajj et al. 2018). These campaigns can serve as models for integrating pharmacists into large-scale diabetes programs that aim to promote novel therapies and improve health literacy (QDA volunteers workshops n.d.).

Within this context, digital health initiatives such as the Wasfaty systems offer some promise to diabetes care through pharmacist-led interventions. Wasfaty provides access and continuity through e-prescribing, ensuring accuracy in dispensing, medication refills, and an efficient medication supply chain, aligning with the Saudi Vision 2030. For patients receiving tirzepatide treatment, these measures can minimize treatment disruption. However, the effectiveness relies heavily on pharmacists' digital literacy, institutional support, and the availability of infrastructure. Variability in these factors across regions contributes to uneven adoption and affects the consistent delivery of pharmacist-led services (Alharthi 2024). The full potential of systems like Wasfaty can only be achieved by expanding the role of clinical pharmacists, enhancing their training, and embedding digital tools into models of care. Such integration is vital for therapies like tirzepatide, where the utilization is not focused only on prescribing but also on pharmacist-led care, which includes monitoring, counselling, and adherence support to diverse patient populations in the region.

### Integration into national diabetes programs

Saudi Arabia has established a robust infrastructure to combat diabetes through its 2020 National Transformation Program (NTP) (National Transformation Program 2020), which aims to improve the quality of care and reduce the economic burden of chronic diseases. Incorporation of tirzepatide into the national diabetes protocols will align with the objectives of NTP by addressing unmet needs in the management of T2D, particularly in high-risk populations. Pharmacists can play a vital role in this integration by acting as accessible healthcare providers who guide patients through the initiation, adjustment, and long-term management of tirzepatide therapy (QDA volunteers workshops n.d.).

The Saudi National Diabetes Centre can benefit from pharmacists' expertise by integrating them into routine care models that coordinate efforts to improve diabetes outcomes through education, research, and clinical management. For example, pharmacists could conduct regular patient reviews to monitor the effectiveness and tolerability of the therapy while providing education on managing potential side effects, such as nausea or appetite changes, commonly associated with incretin-based therapies.

Similarly, the GCC Diabetes Guidelines recommend integrating advanced therapies, such as tirzepatide, into care protocols for high-risk patients. Pharmacists can collaborate with physicians and other healthcare providers to tailor treatment plans to the specific needs of each patient.

## Research gaps in tirzepatide application in the Middle East and Saudi Arabia

Dietary patterns in the Middle East, particularly in Saudi Arabia, have undergone significant changes in the past few decades. High-calorie, low-nutrient diets have become common, contributing to rising obesity rates (Galal 2003). Traditional diets rich in grains, legumes, and fresh produce are being replaced by processed and fast foods, posing a challenge to tailoring medications such as tirzepatide that influence metabolic outcomes.

Middle Eastern populations exhibit unique genetic predispositions to obesity and related diseases such as T2D (Younes et al. 2021; Sherwani et al. 2016). For example, specific genetic variants associated with insulin resistance and obesity are more prevalent in this population, which could influence the efficacy of tirzepatide (Ajabnoor et al. 2023). However, studies directly exploring these genetic interactions with tirzepatide are limited. Obesity in the region is closely linked to socioeconomic status, urbanization, and sedentary lifestyles (Farrag et al. 2017). These factors contribute to the high prevalence of obesity but are not fully addressed in existing tirzepatide trials. Cultural attitudes towards obesity and weight loss differ significantly in the Middle East. The stigma surrounding obesity and the acceptance of larger body sizes in specific communities may affect adherence to pharmacological interventions (Beshyah et al. 2022).

## Areas needing further research

Tirzepatide has been acknowledged to contribute to weight loss, liver health, and reduce cardiovascular risk; however, the underlying biological mechanisms responsible for these benefits have not been completely elucidated. Further research into its impact on fat metabolism, appetite regulation, and inflammatory pathways is needed to clarify these observations. Although short- and medium-term studies suggest that tirzepatide is generally well-tolerated, its long-term safety profile remains incomplete. Gastrointestinal side effects have been the most reported, but the safety of higher doses over extended periods needs further investigation. Concerns exist about potential risks such as pancreatitis or pancreatic cancer, and long-term cardiovascular effects. Ongoing clinical trials and post-marketing surveillance studies are essential to ensure the safe use of tirzepatide.

Beyond safety, understanding how tirzepatide works in the real world, in diverse groups of people, including those with multiple health conditions or limited access to medical care, is crucial.

There is growing interest in co-administering tirzepatide with other treatments, such as SGLT2 inhibitors, statins, or antihypertensives, as they may create synergistic effects by targeting multiple metabolic pathways. This could be particularly beneficial for people with complex conditions such as T2D, obesity, and cardiovascular disease.

Furthermore, research must expand to include specific populations that require distinct treatment strategies. The long-term effects of tirzepatide on diabetic retinopathy and diabetic neuropathy remain a topic of considerable debate. Extended studies are needed to investigate its potential to mitigate or exacerbate these complications over time.

Currently, the safety and efficacy in special populations, such as children, pregnant women, and older adults, remain unclear, requiring further studies.

## Limitations and research gaps

### Thyroid tumors and medullary thyroid carcinoma (MTC)

Short- to intermediate-term data from clinical trials suggest that tirzepatide is generally safe; however, long-term data are still limited. Preclinical studies in rodents showed a potential risk of thyroid C-cell tumors (FDA boxed warning). While the relevance of these findings to humans is unclear, caution is advised (FDA n.d.), particularly in patients with a personal or family history of MTC or Multiple Endocrine Neoplasia syndrome type 2. Emerging data from a few human studies have also found an association between GLP-1 receptor agonists and thyroid malignancies (Madsen et al. 2012). Although the absolute risk is low, continued vigilance is necessary.

### Lack of direct comparison with emerging therapies

Most comparative trials compared tirzepatide against semaglutide at a dose of 1 mg, and head-to-head evaluations with higher doses of semaglutide 2.4 mg (approved for obesity), or with other oral GLP-1 agents are currently lacking. Many existing studies used semaglutide dosages that are lower than those currently approved for T2DM and obesity (Wen et al. 2025). This could limit the understanding of its relative efficacy, safety, and adherence versus these agents. Further studies, especially with longer‐duration trials and varying dosages, can provide insights into the long‐term safety and efficacy of tirzepatide.

### Pancreatitis & retinopathy risk

Reports of pancreatitis and gastrointestinal intolerance, though infrequent, remain notable in real-world settings. Progression of retinopathy was observed in some GLP-1 RA trials, and caution is warranted till further data is available.

### Exclusion of key populations

Current clinical outcomes are mainly derived from RCTs, which exclude patients with advanced renal disease, the elderly, or those with multiple comorbidities. For instance, elderly individuals (≥ 75 years), those with advanced renal impairment (eGFR < 30 mL/min/1.73 m^2^), and individuals with multiple comorbidities were excluded. The representation of racial or ethnic groups was low (Ludvik et al. 2021). These exclusions limit generalizability to real-world populations that are on polypharmacy.

### Economic burden and access in low-resource settings

While modelling suggests significant clinical benefits with the use of tirzepatide, the financial cost of tirzepatide remains substantial. Incremental treatment costs have been estimated to be in the range of $23,563.42 and $47,583.74, depending on the dose (Wang et al. 2025). A budget impact analysis from a health system perspective indicated the use of tirzepatide could result in an additional financial burden ranging from $80 million to $490 million over 5 years on the National Health Insurance System (NHIS) (Zhang and McAdam Marx 2023). Moreover, the incremental cost-effectiveness ratios (ICERs) were estimated at $2,247 per 1% reduction in HbA1c and $237 per additional kilogram of body weight loss. The cost–benefit considerations must be carefully evaluated when expanding access to tirzepatide, particularly in resource-constrained health systems. The need for weekly injections and cold-chain logistics may also limit affordability in settings with limited resources.

### Short trial duration for cardiovascular events

The ongoing SURPASS-CVOT trial (NCT04255433), expected to conclude by 2026, will be pivotal in establishing the definitive role of tirzepatide in cardiovascular risk reduction. Until then, its cardioprotective effects remain promising but not conclusive (Nicholls et al. 2024).

Tirzepatide is efficacious, but uncertainties remain regarding its long-term risks, equitable access, external validity, and cost impact, which can only be resolved through further studies. The potential for rare adverse effects such as thyroid neoplasia remains uncertain (Khurana et al. 2024; Madsen et al. 2012; Abi Zeid Daou et al. 2025) and could be established with the availability of comprehensive data.

## Emerging competitors and future therapies

Tirzepatide represents a significant advancement in T2D and obesity management. Still, the rapidly growing incretin-based therapies that are in the pipeline, including triple agonists and novel combinations, may redefine therapeutic benchmarks.

Cagrilintide (CagriSema), an amylin analogue, is investigated for its potential to support sustained weight loss in overweight or obese individuals, particularly when combined with the GLP-1 receptor agonist semaglutide. Amylin is co-secreted with Insulin from pancreatic beta cells and plays a vital role in promoting satiety by acting on both homeostatic and reward-related regions of the brain. Semaglutide complements this by reducing appetite through GLP-1 receptor activation in the hypothalamus, while also enhancing insulin secretion, suppressing glucagon release, and slowing gastric emptying. These complementary yet distinct mechanisms of action of cagrilintide and semaglutide may emerge as a promising alternative to tirzepatide (D'Ascanio et al. 2024). A study is underway to assess the benefits of combining cagrilintide and semaglutide (ClinicalTrials.gov n.d.).

Retatrutide (LY3437943) is a novel single peptide agonist targeting GIP, GLP-1, and glucagon receptors, conjugated to a fatty diacid moiety to improve its pharmacokinetics. In a phase 1b trial involving individuals with T2D, retatrutide demonstrated dose-dependent reduction in both glycemic parameters and body weight. The highest dose group (12 mg) achieved a placebo-adjusted mean weight loss of approximately 8.96 kg (~ 10%) over 12 weeks, along with improvements in daily plasma glucose (up to −3.1 mmol/L) and sHbA1c (up to −1.6%) (Urva et al. 2022). In phase 2 studies, sustained and significant weight reductions were observed at 24 and 48 weeks across all tested doses (1, 4, 8, and 12 mg) compared to placebo (Jastreboff et al. 2023).

GLP-1 receptor agonists, including dual and triple agonists, have demonstrated efficacy in managing type 2 diabetes (T2D) and obesity. They are also effective in treating NAFLD. Efinopegdutide (MK-6024, HM12525A), a synthetic dual GLP-1/glucagon receptor agonist conjugated to a human IgG Fc via a PEG linker, is one such promising agent. In June 2023, the FDA granted fast-track designation to efinopegdutide for the treatment of NASH. In clinical studies, efinopegdutide achieved a significantly greater reduction in liver fat content compared to the GLP-1 RA semaglutide, with a least square mean relative decrease of 72.7% versus 42.3% at week 24 (Romero-Gómez et al. 2023).

Comparative effectiveness studies and head-to-head trials will be crucial in determining the long-term place of tirzepatide in the treatment algorithm.

## Conclusions

Tirzepatide, a dual GIP and GLP-1 receptor agonist, has the potential to revolutionize the management of metabolic and cardiovascular diseases. Across trials, tirzepatide has demonstrated HbA1c reductions of up to 2.3% and weight loss exceeding 22.5%, positioning it as a highly effective therapy for patients with T2D and obesity. At the same time, SURMOUNT-1 showed that more than 50% of participants achieved a body weight reduction of more than 20%, surpassing the outcomes of existing anti-obesity medications. Beyond glycemic and weight control, tirzepatide holds promise in the management of NASH by reducing hepatic fat content and reversing fibrosis. Additionally, it may play a pivotal role in reducing cardiovascular risk, as it improves lipid profiles, lowers blood pressure, and reduces systemic inflammation, factors that collectively contribute to the prevention of major adverse cardiovascular events. Ongoing studies such as SURPASS-CVOT are expected to provide insights into its cardioprotective potential. Yet, along with these benefits, challenges remain. Limited long-term safety data, potential risks such as thyroid tumors and pancreatitis, high costs, and accessibility challenges, particularly in resource-constrained settings, can limit usage. This focuses on the need for supportive strategies that can maximize benefit and minimize barriers. Pharmacists, as accessible frontline healthcare professionals, play a critical role in enabling patient care by providing education on injection techniques, managing gastrointestinal side effects with dietary advice, addressing medication hesitancy, and ensuring adherence through personalized counselling. Pharmacists can support the use of digital tools, especially among younger populations, to promote long-term adherence. Their interventions help overcome practical barriers related to cost, complexity, or uncertainty, ensuring that patients not only initiate tirzepatide but also persist with it. The transformative potential of tirzepatide lies in its effective integration into patient-centered care. Pharmacists can champion adherence and education, and with continued evaluation of long-term outcomes, tirzepatide may usher in a new era in the fight against obesity, T2D, NASH, and cardiovascular disease.

## Data Availability

All authors confirm all data are available in the main manuscript.
